# Subjective impacts of computerized cognitive training for healthy older adults in the context of the COVID-19 pandemic

**DOI:** 10.1055/s-0043-1767823

**Published:** 2023-04-14

**Authors:** Cristiane Benedita Rodrigues da Mota Antunes Viviani, Tiago Nascimento Ordonez, Andreia Rodrigues Pereira, Karen de Souza Jardim, Jonatas da Hora Borges, Lais Aparecida Pereira Mota, Gabriela dos Santos, Ana Paula Bagli Moreira, Cássia Elisa Rossetto Verga, Graciela Akina Ishibashi, Guilherme Alves da Silva, Luiz Carlos de Moraes, Patrícia Prata Lessa, Beatriz Aparecida Ozello Gutierrez, Sonia Maria Dozzi Brucki, Thais Bento Lima da Silva

**Affiliations:** 1 Universidade de São Paulo, Escola de Artes, Ciências e Humanidades, Departamento de Gerontologia, São Paulo SP, Brazil.; 2 Universidade de São Paulo, Escola de Artes, Ciências e Humanidades, Grupo de Estudos de Treinamento Cognitivo, São Paulo SP, Brazil.; 3 Instituto Supera de Educação, São José dos Campos SP, Brazil.; 4 Universidade de São Paulo, Faculdade de Medicina, Hospital das Clínicas, Grupo de Neurologia Cognitiva e Comportamental, São Paulo SP, Brazil.; 5 Universidade de São Paulo, Faculdade de Medicina, Departamento de Neurologia, São Paulo SP, Brazil.

**Keywords:** Aged, Quality of Life, Cognitive Aging, Digital Technology, Cognition, Idoso, Qualidade de Vida, Envelhecimento Cognitivo, Tecnologia Digital, Cognição

## Abstract

**Background** Computerized cognitive training programs may have benefited the self-assessment of memory, quality of life, and mood among older adults during the coronavirus disease 2019 (COVID-19) pandemic.

**Objective** To determine the subjective impacts of computerized cognitive training on mood, frequency of forgetfulness, memory complaints, and quality of life in the elderly using an online platform.

**Methods** In total, 66 elderly participants of USP 60 + , a program for the elderly offered by Universidade de São Paulo, who voluntarily enrolled in the study were selected and randomized with an allocation ratio of 1:1 into 2 groups: the training group (n = 33) and the control group (n = 33). After signing the free and informed consent form, they answered a protocol which included a sociodemographic questionnaire, the Memory Complaints Questionnaire (MAC-Q), the McNair and Kahn's Frequency of Forgetfulness Scale, the Geriatric Depression Scale (GDS-15), the Geriatric Anxiety Inventory (GAI), and the Control, Autonomy, Self-Realization, and Pleasure (CASP-19) questionnaire. The training cognitive game platform aimed to stimulate various cognitive aspects, including memory, attention, language, executive functions (reasoning, logical thinking), and visual and spatial skills.

**Results** The participants of the training group showed a reduction in the MAC-Q, MacNair and Kahn, and GAI scores in the pre- and posttest comparison. Significant differences were identified between the groups regarding the total scores of the MAC-Q in the post-test, which was also evidenced by the logistic regression.

**Conclusion** Participation in a computerized cognitive intervention promoted reductions in memory complaints, frequency of forgetfulness, and anxiety symptoms, in addition to improving self-reported quality of life.

## Resumo

**Antecedentes** Programas informatizados de treinamento cognitivo podem ter beneficiado a autoavaliação da memória, a qualidade de vida e o humor entre os idosos durante a pandemia de doença do coronavírus 2019 ( *coronavirus disease 2019* , COVID-19, em inglês).

**Objetivo** Determinar os impactos subjetivos do treinamento cognitivo computadorizado no humor, na frequência de esquecimento, nas queixas de memória, e na qualidade de vida em idosos utilizando uma plataforma *online* .

**Métodos** Ao todo, 66 idosos participantes do programa USP 60 + , oferecido à terceira idade pela Universidade de São Paulo, e inscritos voluntariamente no estudo, foram selecionados e randomizados em uma razão de 1:1 em 2 grupos: grupo treinamento (n = 33) e grupo controle (n = 33). Após assinarem o termo de consentimento livre e esclarecido, os participantes responderam a um protocolo que incluía um questionário sociodemográfico, o Questionário de Queixas de Memória (Memory Complaints Questionnaire, MAC-Q), A Escala de Frequência de Esquecimento de McNair e Kahn, a Escala de Depressão Geriátrica (Geriatric Depression Scale, GDS-15), o Inventário de Ansiedade Geriátrica (Geriatric Anxiety Inventory, GAI), e –o questionário de Controle, Autonomia, Autorrealização e Prazer (Control, Autonomy, Self-Realization, and Pleasure, CASP-19). A plataforma de jogos cognitivos de treinamento visou estimular diversos aspectos cognitivos, incluindo memória, atenção, linguagem, funções executivas (raciocínio, raciocínio lógico) e habilidades visuais e espaciais.

**Resultados** Na comparação pré e pós-teste, os participantes do grupo de treinamento apresentaram redução nas pontuações do MAC-Q, da escala McNair e Kahn e do GAI. Diferenças significativas entre os grupos quanto às pontuações totais da escala MAC-Q no pós-teste também foram evidenciadas pela regressão logística.

**Conclusão** A participação em uma intervenção cognitiva computadorizada promoveu reduções nas queixas de memória, frequência de esquecimento e sintomas de ansiedade, além de melhorar a qualidade de vida autorrelatada.

## INTRODUCTION

The coronavirus disease 2019 (COVID-19) pandemic required a return to the literature in search of ways to stimulate the cognition of healthy elderly people who adhered to distancing measures. Consequently, many situations involving interpersonal contact were conducted using information and communication technologies (ICTs). 1

In this scenario, in a systematic review 2 on the use of tablets by the elderly, cognitive benefits were reported in most of the reviewed articles. In one of the studies, 3 which involved 16 elderly people, the authors concluded that electronic devices provided results similar to those of standard cognitive training. In another study 4 testing the effect of the tablet on cognitive skills, which included 22 adults, the results showed that engagement in the intervention was associated with an increase in processing speed and acquisition of new skills. Thus, ICTs have been proven to be useful to improve performance in several different cognitive domains. 2 3 4 5 6 7

However, internet use is also linked to improved well-being, empowerment, autonomy, and independence. 6 Thus, digital inclusion yields different benefits, but cognitive gains have been the focus of a large number of studies. 6 7 During the use of digital tools, many processes and steps can benefit cognition: when the correct procedure to perform a task is remembered, procedural memory is triggered, for example; when tracking the information and actions performed, the working memory is being activated; structuring action in the correct order requires activating executive functions; locating relevant information on the screen requires visual perception; information management takes place to assess what information is relevant; and the attention process is necessary to focus on relevant items, ignoring irrelevant items and stimuli. 6 7

In this context, few studies 8 9 10 have assessed the subjective impacts among the elderly who use such tools and digital platforms. Da Silva et al. 7 highlighted that the combination of cognitive training and other types of interventions to promote health yields multiple benefits to the quality of life of the elderly, such as improvement in metamemory and metacognition, lower risk of frailty, and possible reduction in the risk of dementia. Therefore, the aim of the present study was to determine the impact of computerized cognitive training on mood, frequency of forgetfulness, memory complaints, and quality of life of elderly participants of USP 60 + , a program for the elderly offered by Universidade de São Paulo in partnership with Instituto Supera de Educação.

## METHODS

### Participants

A total of 66 elderly individuals enrolled in the computerized cognitive stimulation workshop offered free of charge by the USP60+ program. They were randomized with an allocation ratio of 1:1 into a training group (n = 33) and a control group (n = 33). In the first semester, the training group received the intervention and, after six months, with the end of the intervention, the control group was able to participate in the online workshop. Comparative statistical tests confirmed that the sociodemographic data of the two groups were similar; therefore, the groups were homogeneous before the start of the intervention. All of them signed the Informed Consent Form and were informed that during the study they could take part in any other study that could potentially have an effect on their cognitive functions.

The sample size was calculated using the G*Power software, version 3.1, from which an alpha significance level of 5% and an effect size of 0.5 were established, with 33 participants in each group, resulting in a power of the sample of 84%.

### Inclusion criteria

The participants were required to be older than 60 years of age and enrolled in USP60 + .

### Exclusion criteria

Individuals under 60 years of age, who were not enrolled in the USP60+ program and did not own a cell phone, tablet or computer with internet access were excluded.

### Supera Online digital platform

**Figure 1:**
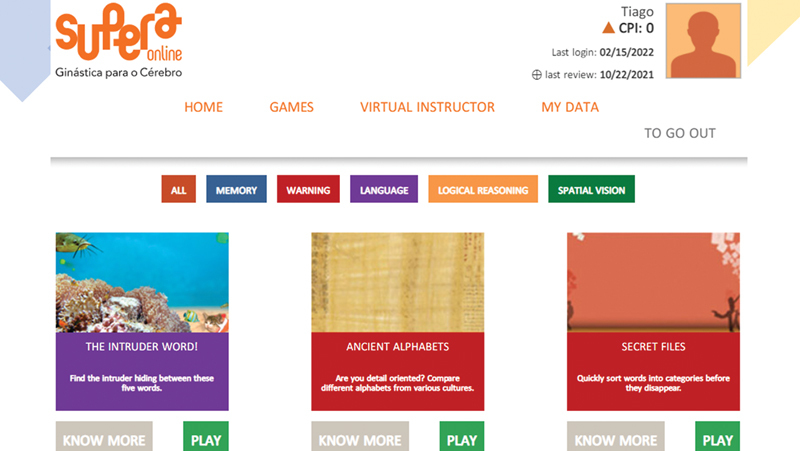
Image from the Supera Online platform.

A French company (HAPPYneuron, Inc., Lyon, France) , through its partner Supera Online (São José dos Campos, SP, Brazil), provided the computerized cognitive stimulation test for 12 weeks for free analysis and evaluation. A web-based cognitive training game platform, it provided participants with a virtual environment with didactic and attractive explanations. Nine exercises developed by Happyneuron for cognitive training were applied for practice during the present study. The brain training cognitive game platform aims to stimulate various cognitive aspects, including memory, attention, language, executive functions (reasoning, logical thinking), and visual and spatial skills, with significant results in several international studies. 11 12 13 14 The platform in Portuguese is available on the following website ( Figure 1 ): www.superaonline.com.br . 15

The program offers training for four categories of users: all age brackets; monitors; coordinators; and managers. Each registered user is assigned a login and password that provide unlimited access to the platform via computers connected to the internet or using mobile devices, such as notebooks, tablets and cell phones. 11 12 13 14 15

In 2016, the Supera Online Digital Platform underwent the process of cultural adaptation and validation for use by mature and older Brazilian adults. The participants in this validation process were 124 healthy mature and older adults who were users of social and health services at the Integrated Center for Health and Education (Centro Integrado de Saúde e Educação, CISE, in Portuguese) in the city of São Caetano do Sul, state of São Paulo. The participants used the platform weekly during 90-minute meetings over a 12-week period. The results of the study, which was conducted and published by Ordonez et al., 6 showed improvements in cognitive performance and mood among the participants.

The current version of the Supera Online training program consists of a digital platform for cognitive stimulation, brain training, and promotion of quality of life. Based on exercises and challenges in the form of digital games, the program develops and stimulates the main cognitive functions: memory, attention, language, logical reasoning, and visuospatial abilities, which contribute to longevity and improved quality of life. 8 9 10

There are twenty modalities of exercises/games grouped into blocks which train specific cognitive abilities: memory, attention, logical reasoning, visuospatial ability and language, which can optimize the cognitive development of participants through play-based interactive activities. The teaching-learning process occurs in a systematized manner, and it is monitored individually by a virtual instructor. Depending on individual progress, users can successively move on to other, more difficult, stages of the tasks programmed on the platform. 8 9 10

### Assessment protocol

#### Sociodemographic questionnaire

Through the sociodemographic questionnaire, we collected data on age, gender, marital status, level of schooling, and healthcare provision.

#### Memory Complaints Questionnaire (MAC-Q)

The Memory Complaints Questionnaire (MAC-Q) is composed of six items related to memory functioning in everyday activities. The higher the score, the greater the severity of the memory-related complaints, and scores ≥ 25 indicate age age-associated impairment, classifying the respondent as having a “negative” memory complaint. 16

#### Frequency of Forgetfulness Scale (McNair and Kahn)

The Frequency of Forgetfulness Scale includes 15 questions on different situations that characterize memory failures, with the following answer options: never (0 points); sometimes (1 point); often (2 points); and always (3 points). The score ranges from 0 to 45 points, with higher scores indicating greater frequency of forgetting. 17

#### Control, Autonomy, Self-realization, and Pleasure (CASP-19) questionnaire

The Control, Autonomy, Self-realization, and Pleasure (CASP-19) questionnaire comprises 19 items measuring perceived quality of life in individuals aged 55 years or older. Each item has 4 response options: never (0 points); not often (1 point); sometimes (2 points); and often (3 points). The score ranges from 0 to 57, and higher scores indicate better perceived quality of life. A total of 6 items (1, 2, 4, 6, 8, and 9) are recorded as negative values and subsequently inverted in the data analysis. 18

#### Geriatric Depression Scale (GDS)

The Geriatric Depression Scale (GDS) quantifies depressive symptoms among the elderly. It consists of a 15-item scale with dichotomous responses: yes or no. Scores < 6 points are defined as normal; from 6 to 10, as mild-to-moderate depression; and > 10, as severe depresssion. 19 20

#### Geriatric Anxiety Inventory (GAI)

The Geriatric Anxiety Inventory (GAI) is used to measure anxiety symptoms; it comprises twenty descriptive statements of anxiety symptoms to be answered subjectively. The total score, which ranges from 0 to 20 points, is the sum of the questions with answers marked as “yes”. 20. Scores higher than 10/11 suggest the presence of generalized anxiety disorder. 21

### Investigation venue

The intervention was carried out at the School of Arts, Sciences and Humanities of Universidade de São Paulo, within a program called Open University for the Elderly, which is currently referred to as USP60 + .

### Data analysis

The Kolmogorov-Smirnov test documented the absence of a normal distribution between continuous and ordinal variables, so we chose to use non-parametric statistics. Therefore, for the descriptive statistics, tables were compiled containing absolute and relative frequencies, as well as measures of position and dispersion (median, interquartile range, minimum and maximum values). The groups were compared using the Chi-squared test for the categorical variables. The Mann-Whitney U test was used for the analysis of the continuous variables, and the Wilcoxon test were used for the analysis of paired samples, 22 23 both followed by the effect size.

Finally, multivariate logistic regression was used to analyze the association regarding the groups and the deltas of the total scores (postest–pretest) of the scales used. All analyses were performed using the Jeffreys's Amazing Statistics Program (JASP, open source), version 0.16.3. The significance level adopted for the statistical tests was of 5%, that is, *p* < 0.05. In addition to the *p* -value, we used the Vovk-Sellke maximum *p* -ratio: based on a two-sided *p* -value, the maximum possible odds in favor of H _1_ over H _0_ . 22 23

### Ethical aspects

The present study was submitted to the Ethics Committee for Research in Humans of the Teaching Hospital of the School of Medicine at Universidade de São Paulo The study was approved under no. 4.357.429.

## RESULTS

Regarding the profile of the 66 older adults interviewed, the sample comprised predominantly female participants, aged between 60 and 92 years, who were married or separated/divorced, and whose level of schooling was higher (39.39%) or postgraduate (42.42%) education. Most of the interviewees had private healthcare insurance and were retired. The participants were divided into two groups (control and training) and, as shown in Tables 1 and 2 , were closely matched statistically in terms of sociodemographic and psychosocial data. The similar profiles enabled the measurement of changes between baseline and postintervention values in the training group.

**Table 1:** Comparison of the sociodemographic data of the study groups

Variable		Total		Training group		Control group		*P* -value
		n = 66	%	n = 33	%	n = 33	%	
Sex	Female	60	90.91	30	90.91	30	90.91	0.937 ^a^
	Male	6	9.09	3	9.09	3	9.09	
Age	Median (interquartile range)	66.00 (6.75)		66.00 (5.00)		66.00 (8.00)		0.893 ^b^
	Minimum-maximum	60.00-92.00		61.00-81.00		60.00-92.00		
Level of schooling	Median (interquartile range)	17.00 (4.00)		17.00 (3.00)		16.00 (5.00)		0.343 ^b^
	Minimum-maximum	8.00-23.00		11.00-22.00		8.00-23.00		
Retired	Yes	62	93.94	32	96.97	30	90.91	0.332 ^a^
	No	4	6.06	1	3.03	3	9.09	

Notes: ^a^ Chi-squared test; ^b^ Mann-Whitney U-Test.

**Table 2:** Comparison of group performance pre- and postintervention

Variables and statistics	General (n = 66)		Training group (n = 33)		Control group (n = 33)		Mann-Whitney test	*P* -value	Effect size	VS-MPR
	Median	IQR	Median	IQR	Median	IQR				
**MAC-Q (pre)**	24.50	4.00	24.00	4.00	25.00	3.00	564.500	0.801	0.037	1.000
**MAC-Q (post)**	24.00	3.75	22.00	6.00	25.00	2.00	266.000	< 0.001	-0.511	137.170
Wilcoxon test	974.000		384.500		114.500					
*P* -value	0.151		0.002		0.123					
Effect size	0.221		0.654		-0.348					
VS-MPR	1.288		32.787		1.427					
**McNair (pre)**	8.50	4.00	9.00	3.00	8.00	4.00	680.500	0.080	0.250	1.820
**McNair (post)**	8.00	4.75	8.00	4.00	9.00	5.00	487.500	0.467	-0.105	1.000
Wilcoxon test	1063.000		377.000		181.500					
*P* -value	0.107		< 0.001		0.441					
Effect size	0.243		0.733		-0.166					
VS-MPR	1.540		96.086		1.000					
**CASP-19 (pre)**	30.50	7.75	32.00	8.00	30.00	5.00	658.500	0.145	0.209	1.315
**CASP-19 (post)**	31.00	6.00	32.00	6.00	30.00	6.00	653.000	0.165	0.199	1.238
Wilcoxon test	794.500		212.500		191.500					
*P* -value	0.496		0.688		0.580					
Effect size	-0.102		-0.086		-0.120					
VS-MPR	1.000		1.000		1.000					
**GDS-15 (pre)**	3.00	2.00	3.00	2.00	2.00	2.00	594.500	0.518	0.092	1.000
**GDS-15 (post)**	2.00	2.75	2.00	2.00	2.00	3.00	531.500	0.870	-0.024	1.000
Wilcoxon test	919.000		1.524		232.00					
*P* -value	0.067		0.124		0.299					
Effect size	0.284		0.342		0.228					
VS-MPR	2.026		1.422		1.019					
**GAI (pre)**	3.00	4.00	3.00	6.00	4.00	3.00	545.500	0.995	0.002	1.000
**GAI (post)**	2.00	4.00	2.00	4.00	2.00	4.00	536.000	0.917	-0.016	1.000
Wilcoxon test	1072.000		275.500		272.500					
*P* -value	0.011		0.037		0.113					
Effect size	0.393		0.458		0.342					
VS-MPR	7.590		3.013		1.495					

Abbreviations: CASP-19, Control, Autonomy, Self-Realization, and Pleasure questionnaire; GAI, Geriatric Anxiety Inventory; GDS-15, Geriatric Depression Scale; IQR, interquartile range; MAC-Q, Memory Complaints Questionnaire; McNair, McNair and Kahn's Frequency of Forgetfulness Scale; VS-MPR, Vovk-Sellke maximum *p* -ratio.

The analysis of the psychosocial data comparing the total scores on the scales showed that the training group scored lower on the MAC-Q, the Frequency of Forgetfulness Scale, and the GAI. Moreover, there was a significant difference between the groups in the total score on the MACQ postintervention ( Table 2 ).

Finally, a multivariate analysis was performed with the aid of logistic regression, in which the groups were categorized (training group = 1; and control group = 0), enabling the association based on the deltas of the total scores (posttest–pretest) of the scales used ( Table 3 ). The logistic regression model generated was statistically significant (χ ^2^ [64] = 14,310; *p* < 0.001), and it correctly classified 73.0% of the cases of participants who had few memory complaints as belonging to the training group.

**Table 3:** Model summary of the logistic regression

Model	Deviance	AIC	BIC	df	ΔΧ ^2^	*P* -value	McFadden R ^2^	Nagelkerke R ^2^	Tjur R ^2^	Cox and Snell R ^2^
1	91.495	93.495	95.685	65			0.000		0.000	
2	77.186	81.186	85.565	64	14.310	< .001	0.156	0.260	0.199	0.195
3	73.694	79.694	86.263	63	3.492	0.062	0.200	0.315	0.244	0.236

Abbreviations: AIC, Akaike Information Criteria; BIC, Bayesian Information Criteria.

Notes: AIC and BIC are measures of fit for the model; the best model will have the lowest AIC and BIC values. Four pseudo-R ^2^ values are calculated in the JASP: McFadden, Nagelkerke, Tjur, and Cox and Snell. These are analogous to R ^2^ in linear regression, and all yield different values. What constitutes a good R ^2^ value varies; however, they are useful when comparing different models for the same data. The model with the largest R ^2^ statistic is considered the best.

**Table 4:** Coefficients of the logistic regression

Model	Parameter	Estimate	Robust standard error	Z	Wald test			VS-MPR
					Wald statistic	df	*P* -value	
1	(Intercept)	0.000	0.246	3,33E-13	1,11E-28	1	1.000	1.000
2	(Intercept)	-0.207	0.290	-0.712	0.536	1	0.476	1.000
	MAC-Q	-0.260	0.085	-3.069	10.179	1	0.002	27.859
3	(Intercept)	-0.268	0.281	-0.952	0.840	1	0.341	1.003
	MAC-Q	-0.225	0.088	-2.559	7.223	1	0.010	7.697
	McNair	-0.155	0.087	-1.774	3.085	1	0.076	1.878

Abbreviations: MAC-Q, Memory Complaints Questionnaire; McNair, McNair and Kahn's Frequency of Forgetfulness Scale; VS-MPR, Vovk-Sellke maximum *p* -ratio.

Note: Training group coded as class 1.

## DISCUSSION

The objective of the present study was to determine the subjective impact of twelve weeks of computerized cognitive training on mood, frequency of forgetfulness, memory complaints, and quality of life of active elderly people enrolled in a program for the elderly in the city from São Paulo. The results of the postintervention assessments and statistical analyses showed improvements in the quality of life of participants in the training group. In addition, there was an improvement in depressive and anxious symptoms, as well as a reduction in memory complaints and related forgetfulness.

These findings corroborate a systematic review 2 on computerized cognitive training, which found a significant improvement in the cognitive and psychological characteristics of healthy older adults. Likewise, the study by Ordonez et al., 6 involving cognitive training with computer games in the elderly, also observed positive effects in the assessment of memory and quality of life.

Thus, the results of the present study pointed to improvements in quality of life, suggesting that training cognitive functions using an online platform can alleviate forgetfulness and memory complaints, leading to improved mental and physical well-being. Menascu et al. 10 suggest that cognitive game training has a beneficial effect on cognitive performance in multiple sclerosis patients with mild cognitive impairment. However, further evaluation is needed to assess the longevity of this effect.

In another review 24 on computerized cognitive training combined with psychoeducation and pen-and-paper-based cognitive training, effects were found for general cognition, working memory, attention, learning, and depressive symptoms; but, in older adults with mild cognitive impairment, there was a lack of effectiveness for non-verbal memory, executive function, processing speed, and visuospatial skills.

Previous studies 6 7 on older patients showed that, in terms of frequency and time of computer use or exposure to different forms of technology, age-related differences were not as great as expected. Among the elderly, individuals showed improvement in some mental skills, including memory, attention, executive functions, and information processing speed. 6 7

In conclusion, the Supera Online Digital Platform offers a range of activities that can help improve cognitive skills, representing an alternative for people with reduced mobility. Other advantages of the program are the lower cost of transport and the convenience of training at home, which contributes to increase the engagement and digital inclusion of the participants. Finally, there is a lack of computerized cognitive training studies with older adults evaluating the subjective effects of these interventions on mood, depression, anxiety, and memory complaints. Future investigations should be conducted to build on the results of the present study.
